# Endothelial Progenitor Cells as a Potential Biomarker in Interstitial Lung Disease Associated with Rheumatoid Arthritis

**DOI:** 10.3390/jcm9124098

**Published:** 2020-12-18

**Authors:** Verónica Pulito-Cueto, Sara Remuzgo-Martínez, Fernanda Genre, Víctor M. Mora-Cuesta, David Iturbe-Fernández, Sonia Fernández-Rozas, Belén Atienza-Mateo, Leticia Lera-Gómez, Pilar Alonso-Lecue, Javier Rodríguez-Carrio, Diana Prieto-Peña, Virginia Portilla, Ricardo Blanco, Alfonso Corrales, Oreste Gualillo, José M. Cifrián, Raquel López-Mejías, Miguel A. González-Gay

**Affiliations:** 1 Research Group on Genetic Epidemiology and Atherosclerosis in Systemic Diseases and in Metabolic Bone Diseases of the Musculoskeletal System, IDIVAL, Santander, 39011 Cantabria, Spain; veronica_pulito_cueto@hotmail.com (V.P.-C.); sara.r.mtz@gmail.com (S.R.-M.); fernandagenre@gmail.com (F.G.); victormanuel.mora@scsalud.es (V.M.M.-C.); david.iturbe@scsalud.es (D.I.-F.); soniam.fernandez@scsalud.es (S.F.-R.); mateoatienzabelen@gmail.com (B.A.-M.); letizialera@hotmail.com (L.L.-G.); alonsolecue@hotmail.com (P.A.-L.); diana.prieto.pena@gmail.com (D.P.-P.); virgiportilla@hotmail.com (V.P.); ricardo.blanco@scsalud.es (R.B.); afcorralesm@hotmail.com (A.C.); josecifrian@gmail.com (J.M.C.); 2 Department of Pneumology, Hospital Universitario Marqués de Valdecilla, Santander, 39008 Cantabria, Spain; 3 López Albo’ Post-Residency Programme, Hospital Universitario Marqués de Valdecilla, Santander, 39008 Cantabria, Spain; 4 Department of Rheumatology, Hospital Universitario Marqués de Valdecilla, Santander, 39008 Cantabria, Spain; 5 Department of Functional Biology, Immunology Area, Faculty of Medicine, Universidad de Oviedo, Oviedo, 33006 Asturias, Spain; javiercarrio@hotmail.com; 6 SERGAS (Servizo Galego de Saude) and IDIS (Instituto de Investigación Sanitaria de Santiago), NEIRID Lab (Neuroendocrine Interactions in Rheumatology and Inflammatory Diseases), Research Laboratory 9, Santiago University Clinical Hospital, Santiago de Compostela, 15706 A Coruña, Spain; oreste.gualillo@sergas.es; 7 School of Medicine, Universidad de Cantabria, Santander, 39005 Cantabria, Spain; 8 Cardiovascular Pathophysiology and Genomics Research Unit, School of Physiology, Faculty of Health Sciences, University of the Witwatersrand, Johannesburg 2050, South Africa

**Keywords:** endothelial progenitor cells, vascular damage biomarker, interstitial lung disease, rheumatoid arthritis, idiopathic pulmonary fibrosis

## Abstract

Interstitial lung disease (ILD) increases morbidity and mortality in patients with rheumatoid arthritis (RA). Although the pathogenesis of ILD associated with RA (RA-ILD^+^) remains poorly defined, vascular tissue is crucial in lung physiology. In this context, endothelial progenitor cells (EPC) are involved in endothelial tissue repair. However, little is known about their implication in RA-ILD^+^. Accordingly, we aimed to investigate the potential role of EPC related to endothelial damage in RA-ILD^+^. EPC quantification in peripheral blood from 80 individuals (20 RA-ILD^+^ patients, 25 RA-ILD^−^ patients, 21 idiopathic pulmonary fibrosis (IPF) patients, and 14 healthy controls) was performed by flow cytometry. EPC were considered as CD34^+^, CD45^low^, CD309^+^ and CD133^+^. A significant increase in EPC frequency in RA-ILD^+^ patients, as well as in RA-ILD^−^ and IPF patients, was found when compared with controls (*p* < 0.001, *p* = 0.02 and *p* < 0.001, respectively). RA-ILD^+^ patients exhibited a higher EPC frequency than the RA-ILD^−^ ones (*p* = 0.003), but lower than IPF patients (*p* < 0.001). Our results suggest that EPC increase may represent a reparative compensatory mechanism in patients with RA-ILD^+^. The degree of EPC frequency may help to identify the presence of ILD in RA patients and to discriminate RA-ILD^+^ from IPF.

## 1. Introduction

Interstitial lung disease (ILD) is one of the most feared complications in patients with rheumatoid arthritis (RA), increasing their morbidity and mortality [1,2]. Although ILD frequently develops in patients with established RA, it may also be presented as the initial or single manifestation of an unidentified RA [1,2,3]. In this regard, the identification of an underlying RA in ILD patients remains a challenge, considering that RA-ILD^+^ shares clinical, pathological, and epidemiological similarities with idiopathic pulmonary fibrosis (IPF), the most common and severe ILD [2,3,4]. Consequently, some patients initially defined as having IPF may be finally diagnosed with RA-ILD^+^ [4]. Based on these data, a better understanding of the pathogenesis of RA-ILD^+^ is crucial.

The pulmonary microvascular endothelium constitutes a large surface area that displays great growth potential [5]. Accordingly, vascular tissue plays a key role in lung homeostasis, contributing both to physiological and pathological processes [5]. In this context, the maintenance of the endothelium through its repair and regeneration is guaranteed by a population of cells described by Asahara et al. and termed as endothelial progenitor cells (EPC) [6]. EPC are mobilized from the bone marrow and possess the capacity to migrate, proliferate, and differentiate into mature endothelial cells stimulating the formation of new blood vessels [6]. Interestingly, accumulating evidence has shown the implication of EPC in several diseases, although controversial results have been reported in RA [7,8,9,10] and IPF [11,12]. As far as we know, the specific role of EPC in the pathogenesis of RA-ILD^+^ remains largely unknown. Given that EPC have been suggested as a marker of endothelial damage in several diseases, the understanding of the role of EPC in RA-ILD^+^ may be critical for a better knowledge of the vascular pathophysiology that characterizes this disease.

Taking these considerations into account, the aim of this study was to investigate the potential role of EPC in the pathogenesis related to endothelial damage in RA-ILD^+^. For this purpose, we compared the frequency of EPC in patients with RA-ILD^+^ with that observed in the following three groups: RA-ILD^−^ patients, IPF patients, and healthy controls. In addition, the association of EPC frequency with the demographic and clinical features of these patients was determined.

## 2. Materials and Methods

### 2.1. Study Population

A total of 80 individuals, constituted by 20 RA-ILD^+^ patients and three comparative groups (25 RA-ILD^−^ patients, 21 IPF patients, and 14 healthy controls), were recruited from the Pneumology and Rheumatology departments of Hospital Universitario Marqués de Valdecilla (Santander, Spain). RA-ILD^−^ patients fulfilled the 2010 American College of Rheumatology criteria for RA [13]. ILD was excluded in RA-ILD^−^ patients by the evaluation of high-resolution computed tomography (HRCT) images of the chest by experienced radiologists. Those who fulfilled the American Thoracic and European Respiratory Society’s criteria for ILD were classified as RA-ILD^+^ [14]. IPF patients met their own criteria [14]. Healthy controls did not present any history of autoimmune or lung diseases.

Demographic and clinical features of patients comprising sex, age, smoking history, duration of RA disease, rheumatoid factor (RF) and anti-cyclic citrullinated peptide antibodies (ACPA) status, C-reactive protein (CRP), erythrocyte sedimentation rate (ESR), pulmonary function tests (PFTs), HRCT pattern, and immunosuppressive therapies received by RA patients at the time of the study were collected. HRCT patterns of ILD patients were stratified according to the criteria for the usual interstitial pneumonia (UIP) pattern of the Fleischner Society [15].

All the experiments involving humans and human blood samples were carried out in accordance with the approved guidelines and regulations, according to the Declaration of Helsinki. All experimental protocols were approved by the Ethics Committee of clinical research of Cantabria, Spain (2016.092). All subjects gave written informed consent to participate in this study previous to their inclusion.

### 2.2. EPC Quantification by Flow Cytometry

EPC from peripheral venous blood were characterized by simultaneous expression of cell surface markers that reflect stemness (CD34), immaturity (CD133), endothelial commitment (CD309 or vascular endothelial growth factor receptor 2), and a low expression of the pan-leukocyte marker (CD45) [16,17,18].

EPC quantification was analysed by direct flow cytometry, following the recommendations on EPC measurement that were previously described [16,17,18]. Briefly, 200 µL of peripheral blood was pre-incubated with Fc receptors blocking reagent (Miltenyi Biotech, Madrid, Spain). Then, cells were labelled with APC-conjugated anti-CD34 (Miltenyi Biotech, Madrid, Spain), VioBright FITC-conjugated anti-CD309 (VEGFR-2) (Miltenyi Biotech, Madrid, Spain), PE-conjugated anti-CD133/2(293C3) (Miltenyi Biotech, Madrid, Spain), Vioblue-conjugated anti-CD45 (Miltenyi Biotech, Madrid, Spain) monoclonal antibodies, or with isotype-matched antibodies (Miltenyi Biotech, Madrid, Spain). After conjugation, red blood cells were lysed by incubating in flow cytometry and fluorescence-activated cell sorting (FACS) lysing solution (BD Bioscience, San Jose, CA, USA) and white blood cell pellets were then washed once with phosphate-buffered saline (PBS). Labelled cells were analysed in a CytoFLEX flow cytometer (Beckman Coulter, Brea, CA, USA) using a Cytexpert 2.3 analyzer (Beckman Coulter, Brea, CA, USA), acquiring approximately 1 × 10^5^ per sample. First, CD34^+^ and CD45^low^ were gated and then assayed for expression of CD133 and CD309 in the lymphocyte gate. Thus, EPC were considered as CD34^+^, CD45^low^, CD133^+^ and CD309^+^ cells. EPC quantification was expressed as the percentage of cells in the lymphocyte gate.

### 2.3. Statistical Analyses

Data were expressed as mean ± standard deviation (SD) for continuous variables, and number of individuals (*n*) and percentage (%) for categorical variables. The differences between RA-ILD^+^ and RA-ILD^-^ patients in sex, smoking status, RF and ACPA status, as well as in therapies received were analysed by chi-square test, whereas the differences in age at study, duration of RA disease, CRP and ESR levels and PFTs were determined by Student´s t-test. Comparisons of EPC frequency between two study groups were performed by Student’s t-test. Relationship of EPC frequency with continuous variables and categorical variables related to disease features was carried out via estimation of the Pearson’s correlation coefficient (r) and one-way ANOVA, respectively. *p*-values <0.05 were considered as statistically significant. Statistical analysis was performed using STATA statistical software 12/SE (Stata Corp., College Station, TX, USA).

## 3. Results

### 3.1. Demographic and Clinical Features of Patients and Controls

The mean age ± SD at the time of the study of healthy controls, RA-ILD^+^, RA-ILD^−,^ and IPF patients was 41.8 ± 13.7, 66.8 ± 10.2, 60.1 ± 11.8 and 69.2 ± 10.0 years, respectively. Additionally, information on sex and smoking status of individuals, as well as clinical features, is displayed in Table 1.

As expected, statistically significant differences were found between patients with RA-ILD^-^ and RA-ILD^+^ regarding the following clinical features: RF and ACPA status, CRP levels, forced expiratory volume in one second /forced vital capacity (FEV1/FVC) (% predicted), diffusing capacity of the lung for carbon monoxide (DLCO) (% predicted), and therapies received (Table 1).

### 3.2. Differences of EPC Frequency between RA-ILD^+^ Patients and the Comparative Groups

**Figure 1:**
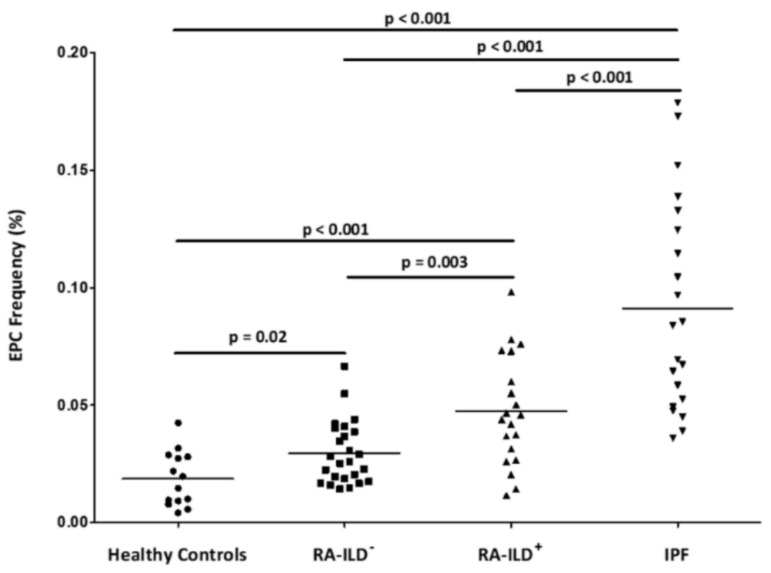
Quantification of EPC population by flow cytometry in all individuals included in the study. EPC were considered as CD34 ^+^, CD45^low^, CD309^+^ and CD133^+^ cells in the lymphocyte gate and were expressed as the percentage of cells in this gate. Differences between the study groups were evaluated by Student’s t-test. Horizontal bars indicate the mean value of each study group. *p: p-*value; EPC: endothelial progenitor cells; RA: rheumatoid arthritis; ILD: interstitial lung disease; IPF: idiopathic pulmonary fibrosis.

A significant increase in EPC frequency in patients with RA-ILD^+^ was found when compared with healthy controls (*p* < 0.001) (Figure 1 and Supplementary Table S1).

Furthermore, EPC frequency in patients with RA-ILD^+^ was also significantly different from RA-ILD^−^ and IPF patients (Figure 1 and Supplementary Table S1). In particular, patients with RA-ILD^+^ exhibited a higher EPC percentage than the RA-ILD^−^ ones (*p* = 0.003), but lower than IPF patients (*p* < 0.001) (Figure 1 and Supplementary Table S1).

In addition, patients with RA-ILD^−^ and IPF showed a higher frequency of EPC than healthy controls (*p* = 0.02 and *p* < 0.001, respectively) (Figure 1). Moreover, patients with RA-ILD^−^ revealed a lower frequency of EPC than IPF patients (*p* < 0.001) (Figure 1).

### 3.3. Relationship of EPC Frequency with Clinical Features

No correlation of EPC frequency with the duration of RA disease, CRP, and ESR was observed in RA patients, regardless of the presence or absence of underlying ILD (Table 2). This was also the case when the relationship of EPC frequency with PFTs of RA-ILD^+^, RA-ILD^−^, and IPF patients was analyzed (Table 2). Likewise, we could not observe differences in the EPC frequency when RA-ILD^+^ patients were stratified according to smoking history, RF/ACPA status, HRCT pattern, or therapies (Table 3). Additional information on RA-ILD^−^ and IPF patients is shown in Table 3.

Since IPF and RA-ILD^+^ with UIP share a similar HRCT pattern, we assessed if differences in the frequency of EPC between them might exist. In this regard, we observed that the frequency of EPC was higher in IPF than in RA-ILD^+^ with UIP pattern (*p* < 0.001).

## 4. Discussion

EPC are crucial in several inflammatory diseases in which endothelial damage plays a fundamental role [7,8,9,10,11,12,16,19,20,21]. In this line, and to the best of our knowledge, no data regarding the role of EPC in RA-ILD^+^, a severe complication in RA patients, were available until the present study. Accordingly, the purpose of this work was to determine the potential role of EPC in the pathogenesis related to endothelial damage in RA-ILD^+^.

Our results show, for the first time, a higher EPC frequency in the peripheral blood of RA-ILD^+^ patients when compared with healthy controls, supporting an increase in EPC production and mobilization into the systemic circulation. In this regard, we hypothesize that EPC are being recruited at the sites of vascular damage to exert their repair capacities as a compensatory mechanism related to endothelial damage in RA-ILD^+^ patients. In addition, and in accordance with previous studies in RA and IPF [7,11], our data also revealed a significant increase in EPC frequency in RA-ILD^−^ and IPF patients in relation to controls, further supporting the compensatory mechanism of these cells.

Interestingly, we also disclosed, for the first time, a significant difference in the EPC frequency of RA-ILD^+^ patients when compared with IPF and RA-ILD^−^ patients. In keeping with that, patients with the most severe ILD, represented by IPF patients, showed the highest frequencies of EPC. Accordingly, patients with RA-ILD^+^ exhibited a higher frequency of EPC than those patients with RA-ILD^−^ who did not present any ILD. This suggests a potential value of the quantification of EPC as a complementary tool for establishing a differential diagnosis between these diseases.

Regarding the association of EPC frequency with disease features, no relationship was observed. In agreement with this, previous reports showed a lack of association of EPC quantification with PFTs and smoking status in IPF patients [12], as well as with the duration of RA disease, CRP and ESR levels, ACPA and RF status, or therapies in RA patients [7,8].

We acknowledge that the present study has some limitations. The difference in the age between patients and controls may constitute a weakness of our study. However, whereas significant differences in the frequency of EPC existed between RA-ILD^+^ and RA-ILD^−^, the age was similar between these two subgroups of RA. It was also applicable when RA patients, regardless of their ILD status, were compared with those with IPF. In addition, previous studies have shown that age does not constitute a relevant influence on the number of EPC in peripheral blood in healthy controls [22]. Furthermore, statistical differences in some clinical characteristics between RA groups, especially DLCO (% predicted), may also constitute a potential limitation of our study.

In conclusion, our results suggest that EPC increase may represent a reparative compensatory mechanism in patients with RA-ILD^+^. Moreover, clinical assessment of EPC frequency as a biomarker of endothelial damage may help to identify the presence of ILD in patients with RA and to discriminate RA-ILD^+^ from IPF.

The results of this work were partially presented at the 2019 American College of Rheumatology Meeting in Atlanta, Georgia, USA (abstract no. 57) (View Abstract and Citation Information Online- https://acrabstracts.org/abstract/endothelial-progenitor-cells-in-the-pathophysiology-of-interstitial-lung-disease-associated-with-rheumatoid-arthritis/) and at the 2020 European E-Congress of Rheumatology (abstract no. SAT0014) (View Abstract and Citation Information Online- https://eular.conference2web.com/#!resources/endothelial-progenitor-cells-role-in-endothelial-damage-of-interstitial-lung-disease-associated-to-rheumatoid-arthritis).

## Figures and Tables

**Table 1:** Demographic and clinical features of the individuals included in this study.

	Healthy Controls *n* = 14	RA-ILD^+^ Patients *n* = 20	RA-ILD^−^ Patients *n* = 25	IPF Patients *n* = 21	* p
Sex (women), *n* (%)	7 (50)	9 (45.0)	15 (60.0)	7 (33.3)	0.32
Age at study, mean ± SD, years	41.8 ± 13.7	66.8 ± 10.2	60.1 ± 11.8	69.2 ± 10.0	0.05
Smoking ever, *n* (%)	3 (27.3)	13 (65.0)	13 (52.0)	16 (76.2)	0.38
Duration of RA disease, mean ± SD, years	−	9.2 ± 10.2	4.1 ± 7.4	−	0.06
RF positive, *n* (%)	−	16 (80.0)	11 (44.0)	−	**0.01**
ACPA positive, *n* (%)	−	18 (90.0)	15 (60.0)	−	**0.02**
CRP (mg/dL), mean ± SD	−	1.1 ± 1.1	0.5 ± 0.5	−	**0.04**
ESR (mm/1st hour), mean ± SD	−	22.8 ± 27.2	14.4 ± 12.4	−	0.24
**Pulmonary function tests**					
FVC (% predicted), mean ± SD	−	95.1 ± 24.7	99.2 ± 16.0	84.9 ± 14.7	0.58
FEV1 (% predicted), mean ± SD	−	91.7 ± 21.5	94.9 ± 22.0	87.3 ± 19.6	0.67
FEV1/FVC (% predicted), mean ± SD	−	77.6 ± 9.3	93.6 ± 12.3	79.7 ± 7.8	**<0.001**
DLCO (% predicted), mean ± SD	−	40.9 ± 13.9	79.9 ± 20.0	43.6 ± 18.4	**<0.001**
**HRCT pattern**					
UIP pattern, *n* (%)	−	11 (55.0)	−	21 (100.0)	−
Probable UIP pattern, *n* (%)	−	1 (5.0)	−	−	−
NSIP pattern, *n* (%)	−	7 (35.0)	−	−	−
Non-NSIP pattern, *n* (%)	−	1 (5.0)	−	−	−
**Therapies received by RA patients**					
csDMARDs, *n* (%)	−	17 (85)	13 (52)	−	**0.02**
bDMARDs, *n* (%)	−	15 (75)	2 (8)	−	**<0.001**

RA: rheumatoid arthritis; ILD: interstitial lung disease; IPF: idiopathic pulmonary fibrosis; SD: standard deviation; RF: rheumatoid factor; ACPA: anti-cyclic citrullinated peptide antibodies; CRP: C-reactive protein; ESR: erythrocyte sedimentation rate; FVC: forced vital capacity; FEV1: forced expiratory volume in one second; DLCO: diffusing capacity of the lung for carbon monoxide; HRCT: high resolution computed tomography; UIP: usual interstitial pneumonia; NSIP: non-specific interstitial pneumonia; csDMARDs: conventional synthetic disease-modifying anti-rheumatic drugs; bDMARDs: biologic disease-modifying anti-rheumatic drugs. * *p*: *p-*value obtained after comparison between RA-ILD^+^ and RA-ILD^−^ patients. Statistically significant results are highlighted in bold.

**Table 2:** Correlation of EPC frequency with continuous variables related to disease features.

	RA-ILD^+^ Patients		RA-ILD^−^ Patients		IPF Patients	
	*r*	*p*	*r*	*p*	*r*	*p*
Duration of RA disease (years)	−0.19	0.42	−0.04	0.87	−	−
CRP (mg/dL)	−0.06	0.79	−0.30	0.20	−	−
ESR (mm/1st hour)	−0.34	0.10	−0.06	0.82	−	−
FVC (% predicted)	0.07	0.76	0.26	0.34	−0.21	0.35
FEV1 (% predicted)	−0.07	0.77	0.29	0.29	−0.19	0.40
FEV1/FVC (% predicted)	−0.03	0.89	0.24	0.40	−0.05	0.83
DLCO (% predicted)	0.38	0.22	0.07	0.81	−0.23	0.40

EPC: endothelial progenitor cells; RA: rheumatoid; ILD: interstitial lung disease; IPF: idiopathic pulmonary fibrosis; *r*: Pearson’s correlation coefficient; *p*: *p*-value; CRP: C-reactive protein; ESR: erythrocyte sedimentation rate; FVC: forced vital capacity; FEV1: forced expiratory volume in one second; DLCO: diffusing capacity of the lung for carbon monoxide.

**Table 3:** Differences in EPC frequency according to categorical variables related to disease features.

Variable	Category	RA-ILD+ Patients		RA-ILD− Patients		IPF Patients	
		*Mean ± SD*	*p*	*Mean ± SD*	*p*	*Mean ± SD*	*p*
Smoking ever	No	0.059 ± 0.020	0.20	0.028 ± 0.010	0.53	0.092 ± 0.048	0.93
	Yes	0.043 ± 0.024		0.031 ± 0.016		0.091 ± 0.045	
RF	No	0.061 ± 0.034	0.31	0.026 ± 0.009	0.18	−	−
	Yes	0.047 ± 0.020		0.034 ± 0.017		−	
ACPA	No	0.046	0.88	0.026 ± 0.009	0.34	−	−
	Yes	0.049 ± 0.023		0.031 ± 0.015		−	
HRCT pattern	UIP	0.043 ± 0.020	0.15	−	−	0.091 ± 0.045	−
	NSIP	0.059 ± 0.025		−		−	
csDMARDs	No	0.036 ± 0.099	0.39	0.033 ± 0.016	0.25	−	−
	Yes	0.049 ± 0.025		0.027 ± 0.011			
bDMARDs	No	0.047 ± 0.034	0.99	0.030 ± 0.014	0.31	−	−
	Yes	0.047 ± 0.020		0.020 ± 0.004			

EPC: endothelial progenitor cells; RA: rheumatoid arthritis; ILD: interstitial lung disease; IPF: idiopathic pulmonary fibrosis; SD: standard deviation; *p*: *p*-value; RF: rheumatoid factor; ACPA: anti-cyclic citrullinated peptide antibodies; HRCT: high resolution computed tomography; UIP: usual interstitial pneumonia; NSIP: non-specific interstitial pneumonia; csDMARDs: conventional synthetic disease-modifying anti-rheumatic drugs; bDMARDs: biologic disease-modifying anti-rheumatic drugs.

## Supplementary Materials

The following are available online at https://www.mdpi.com/2077-0383/9/12/4098/s1, Table S1: Differences of EPC frequency between RA-ILD^+^ patients and the three comparative groups (healthy controls, RA-ILD^−^ and IPF patients).
