# Supplementary material for: Endothelial Progenitor Cells as a Potential Biomarker in Interstitial Lung Disease Associated with Rheumatoid Arthritis
Source: J Clin Med. 2020 Dec 18;9(12):4098. doi: 10.3390/jcm9124098 (PMC7766338; doi:10.3390/jcm9124098)
Supplement: Supplementary file 1 [file jcm-09-04098-s001.pdf]

Supplementary

Table S1. Differences of EPC frequency between RA-ILD$^+$ patients and the three comparative groups (healthy controls, RA-ILD$^-$ and IPF patients).

	**EPC Frequency (Mean ± SD)**	***p***
RA-ILD$^+$ patients vs healthy controls	0.047 ± 0.023 vs 0.019 ± 0.012	**< 0.001**
RA-ILD$^+$ patients vs RA-ILD$^-$ patients	0.047 ± 0.023 vs 0.030 ± 0.013	**0.003**
RA-ILD$^+$ patients vs IPF patients	0.047 ± 0.023 vs 0.091 ± 0.045	**< 0.001**

EPC: endothelial progenitor cells; RA: rheumatoid arthritis; ILD: interstitial lung disease; IPF: idiopathic pulmonary fibrosis; SD: standard deviation. Significant results are highlighted in bold.
